# A rare presentation of pheochromocytoma in pregnancy: a case report

**DOI:** 10.1186/s13256-017-1549-z

**Published:** 2018-02-09

**Authors:** Seyedeh Noushin Ghalandarpoor-Attar, Seyedeh Mojgan Ghalandarpoor-Attar, Sedigheh Borna, Fahimeh Ghotbizadeh

**Affiliations:** 1 Obstetrics and Gynecology Department, Imam Khomeini Hospital, Tehran University of Medical Sciences, Tehran, Iran; 2 Obstetrics and Gynecology Department, Baharloo hospital, Tehran University of Medical Sciences, Tehran, Iran; 3 Valiasr Hospital, Imam Khomeini Hospital Complex, East Baqerkhan st, Chamran Highway, Tehran, 1419733141 Iran

**Keywords:** Hypertension, Pregnancy, Pheochromocytoma

## Abstract

**Background:**

Early diagnosis of pheochromocytoma and its proper management can lessen its mortality and morbidity. This case report describes a 24-year-old pregnant woman with an unusual presentation of pheochromocytoma.

**Case presentation:**

An Iranian 24-year-old primigravid woman from Kordistan province was referred to our center with left flank pain at 37 weeks of gestation. She had a history of gestational diabetes mellitus since the 12th week of gestation which was managed by insulin administration. She also had a history of pulsatile bi-temporal headache for 2 years prior to her referral to us. She underwent complete abdominal and pelvic ultrasound imaging for her flank pain. This examination revealed a heterogeneous mass of 119 × 87 × 79 mm above her left kidney, highly suspicious of being an adrenal-originating tumor. Subsequently, we consulted an endocrinologist. She underwent abdominopelvic magnetic resonance imaging and her 24-hour urine metanephrine, normetanephrine, and vanillylmandelic acid were assessed. Finally, the diagnosis of pheochromocytoma was confirmed. She underwent a cesarean section and adrenal mass excision at the 40th week of gestation. This timely diagnosis resulted in her proper management and good maternal and neonatal treatment outcomes.

**Conclusions:**

Our patient had pheochromocytoma during pregnancy. She had no complaints about hypertension before or during pregnancy until giving birth to her child; her only symptoms were a vague left flank pain, gestational diabetes, and headaches for the past 2 years. The unusual symptom of flank pain led to timely diagnosis and a good treatment outcome.

## Background

Pheochromocytoma is a catecholamine-secreting tumor originating from chromaffin cells of medullary region of adrenal gland or sympathetic ganglia. Pheochromocytoma accounts for 0.2 to 0.4% of hypertensive disorders and it may lead to severe or lethal hypertensive crisis [1, 2]. Its prevalence during pregnancy is approximately 0.0002% [3, 4]. Timely diagnosis of this disease and its proper management can lessen maternal and fetal mortality and morbidity from around 50% to less than 5% and 15%, respectively [5–10].

During pregnancy pheochromocytoma might not have any specific signs and symptoms. It is less prevalent than other hypertensive disorders during pregnancy. Also specific symptoms such as paroxysmal attacks of sweating, palpitation, and blood pressure crisis are probably lower among pregnant women compared to non-pregnant woman [8]. A literature review shows that 90% of pregnant women demonstrate symptoms of pheochromocytoma right before delivery [8]. Thus, this may result in delayed diagnosis, putting the mother and child at risk.

## Case presentation

An Iranian 24-year-old primigravid woman from Kordistan province presented to our hospital with left flank pain. She had had the pain for a few days and had received adequate prenatal care until her referral to us. She was diagnosed as having had gestational diabetes mellitus since the 12th week of gestation (her fasting blood sugar was between 106 and 116 mg/dL). Insulin therapy was started at a dosage of 4 units of neutral protamine Hagedorn at bedtime. All fasting and postprandial blood sugars had been controlled well.

She also complained of having had pulsatile bi-temporal headaches for 2 years prior to her referral to us. Her headaches responded well to common analgesics. She also mentioned that she had hypertension of 140/90 mmHg at one of her prenatal visits during the 32nd week of gestation. However, the physician had not diagnosed hypertension by rechecking her blood pressure at that time. Although her gynecologist had assessed proteinuria via 24-hour urine sample, no proteinuria was detected (119 mg/dL). Her liver enzyme, creatinine, and platelet counts were all in normal range.

At the 37th week of gestation she had presented to us with left flank pain and no urinary symptoms. We did not detect costovertebral tenderness, fever, hypertension, or tachycardia at the primary examinations. Urine analysis was completely normal, so we did ultrasound imaging of fetus and kidneys. Ultrasound imaging revealed no problems with her kidneys, but we found a solid mass with fine cystic component of 119 × 87 × 79 mm above her kidney, highly suspicious for an adrenal-originating tumor. The fetus had no abnormality at the ultrasound imaging.

**Fig. 1:**
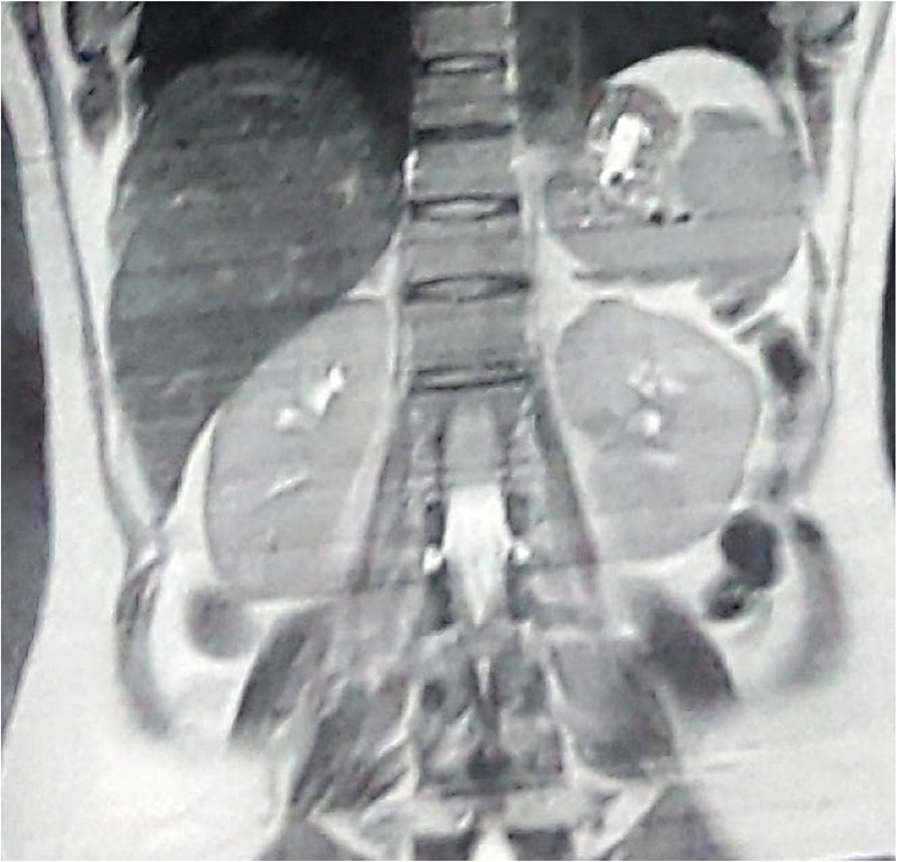
Magnetic resonance imaging showing a well-defined 100 × 95 mm heterogeneous mass in the left adrenal region above the kidney

To evaluate the mass, we consulted with an endocrinologist to definitely diagnose the incidentaloma. Abdominopelvic magnetic resonance imaging without contrast showed a well-defined 100 × 95 mm heterogeneous mass in her left adrenal region above the kidney which contained some cystic areas and showed some restriction foci on diffusion-weighted magnetic resonance imaging (Fig. 1). No obvious signal drop was seen on the opposed phase image. Other organs seemed intact. Her 24-hour urine metanephrine, normetanephrine, and vanillylmandelic acid were assessed (Table 1).

**Table 1:** Our patient’s 24-hour urine analysis

24 hours urine	Results	Normal range
Volume	1100 mL/day	600–2500
Creatinine	726 mg/day	600–1800
Protein	44 mg/day	<150
Calcium	132 mg/day	< 250
Metanephrine	510 μg/day	< 530
Normetanephrine	756 μg/day	< 600
Vanillylmandelic acid	1.2 mg/day	2.05–4.25

**Fig. 2:**
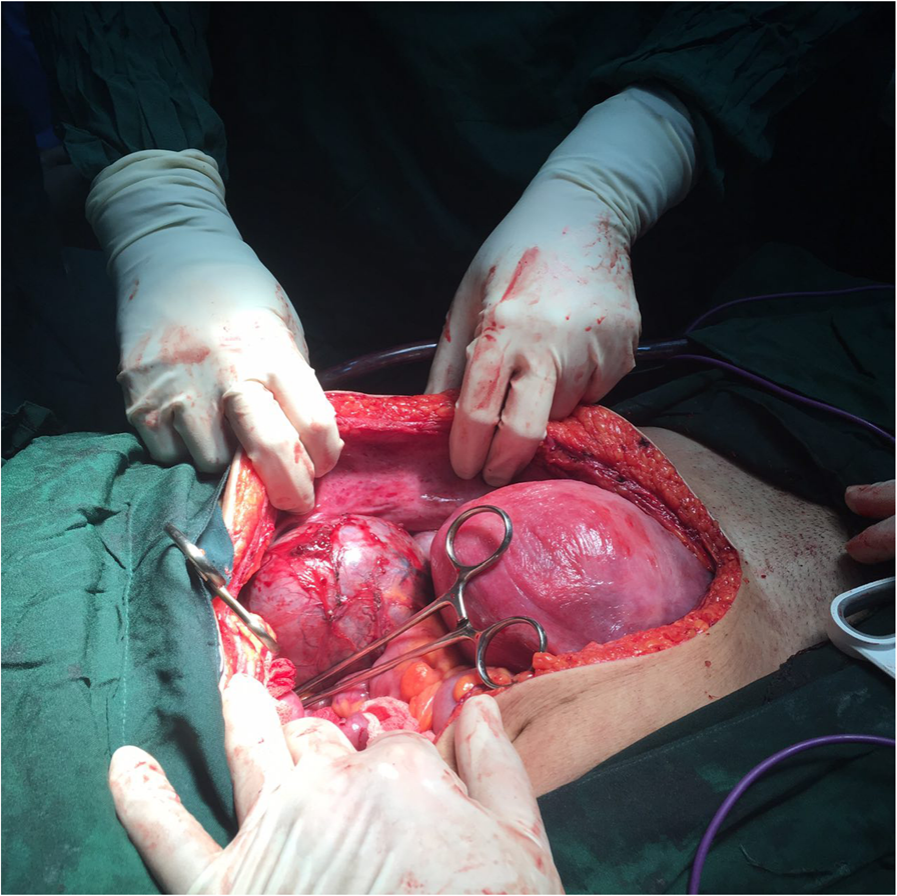
Intraoperative view showing a huge mass in left adrenal region

All workups were done according to association between pheochromocytoma and multiple endocrine neoplasia syndrome. Thyroid-stimulating hormone and ultrasound imaging of her thyroid and parathyroid glands were normal. She had no dermatitis in her family history. After confirming the diagnosis of pheochromocytoma, she was transferred to our endocrinology ward and treatment with phenoxybenzamine was started. Atenolol was added because she showed one hypertensive crisis (150/100 mmHg). Ten days after the beginning of medical treatment she underwent laparotomy and cesarean section. After induction of anesthesia in the operation room, her blood pressure rose to 240/180 mmHg. Blood pressure fluctuations were managed by intravenously administered nitroprusside. First, a cesarean was done and a boy weighing 3300 grams was born. However, he had secondary apnea after some minutes. This was managed by positive pressure ventilation and he responded well. After repairing uterine incision, endocrine surgeons began tumor resection, which measured approximately 12 × 13 cm in diameter, but no surrounding organ invasion was detected (Fig. 2). She was taken to our intensive care unit after the surgery. Her hemodynamic condition was monitored for 24 hours. She was discharged after 3 days while all her vital signs were normal.

## Discussion

Pheochromocytoma is rare among hypertensive patients especially during pregnancy. It is most common in the fourth to fifth decades of life in both men and women [1, 2]; however, our patient was just 24-years old.

Diagnosis of pheochromocytoma in pregnant women is often hard and the presence of hypertension can be deceptive. The patient might have no symptoms until delivery time [11]. Persistent or paroxysmal hypertension is the most common sign of this disease and only 5 to 15% of patients are normotensive [12–14]. Headache (mild to severe) occurs in up to 90% of symptomatic patients [14]. An abnormality in carbohydrate metabolism may occur because of increased catecholamines secretion [15].

Our patient was diagnosed as having pheochromocytoma after we evaluated her vague unilateral flank pain. The other rare presentation of pheochromocytoma in our case was the early onset of insulin-dependent diabetes mellitus. Of interest, her blood pressure was normal until near delivery when she had an episode of rising blood pressure to 150/100 mmHg. Headache is a symptom of pheochromocytoma with different durations and severity. Our patient had had headaches for 2 years prior to her referral to us, but her neurological examination was normal. It seems that since having a headache is common in pregnancy and the severity of the headache had not changed during her conception, her gynecologist had not asked for additional evaluation. Hence, it may be reasonable to consider the chances of pheochromocytoma when a pregnant woman has early onset of diabetes mellitus plus other related symptoms such as headache or flank pain.

Our patient had gestational diabetes mellitus in the first trimester and an episode of hypertension at one of her prenatal visits. So, if the presence and severity of headache was reassessed at most of her prenatal visits and a neurologist was consulted, maybe pheochromocytoma could have been one of her differential diagnoses. In this way, the diagnostic workup could have led to an earlier diagnosis.

Some factors can cause clinically overt pheochromocytoma such as increase in intraabdominal pressure, fetal movement, uterine contraction, delivery (vaginal or surgical), and even general anesthesia [5, 16, 17]. The main goal of pheochromocytoma management is preventing hypertension crisis. Thus, medical treatment with alpha blocker should be initiated at the time of diagnosis and continued for at least 10 to 14 days before surgery [18]. Phenoxybenzamine is the drug of choice (pregnancy class c). This drug can cross the placenta and reach the fetus. It may cause perinatal depression and transient hypotension in the neonate [19], as seen in our patient’s neonate.

Surgery is the definite treatment of pheochromocytoma, but there is controversy about the appropriate time. The related factors are gestational age, tumor accessibility, presence or absence of fetal distress, and patient’s response to medical treatment [20]. Some authors recommend tumor resection as soon as adequate medical treatment was done. If the tumor is less than 7 cm in diameter, laparoscopic surgery can be done. But if the pregnant woman is in the 24th week of gestation or more, surgical removal is delayed until time of cesarean delivery [20, 21] as we did for our patient.

## Conclusions

Although pheochromocytoma is rare among hypertensive patients especially during pregnancy, our case demonstrated that early diagnosis and close collaboration of gynecologist, hypertension specialist, endocrinologist, anesthesiologist, and pediatrician can result in good maternal and fetal treatment outcomes. However, delay in diagnosis could result in catastrophic results. In our case the patient had severe paroxysmal hypertension after anesthesia induction.
